# Dissatisfaction with dentofacial appearance and the normative need for orthodontic treatment: determinant factors

**DOI:** 10.1590/2176-9451.19.3.120-126.oar

**Published:** 2014

**Authors:** Anderson Barbosa de Almeida, Isabel Cristina Gonçalves Leite, Camilo Aquino Melgaço, Leandro Silva Marques

**Affiliations:** 1 MSc in Collective Health, Federal University of Juiz de Fora, UFJF.; 2 Adjunct professor, Federal University of Juiz de Fora, UFJF.; 3 Professor, School of Dentistry - Federal University of Minas Gerais, UFMG.; 4 Adjunct professor, Federal University of the Jequitinhonha and Mucuri Valleys, UFVJM.

**Keywords:** Malocclusion, Orthodontics, Quality of life

## Abstract

**Objective:**

This study aims at assessing the normative need for orthodontic treatment and the factors that determine the subjective impact of malocclusion on 12-year-old Brazilian school children.

**Methods:**

A total of 451 subjects (215 males and 236 females) were randomly selected from private and public schools of Juiz de Fora, Brazil. The collected data included sociodemographic information and occlusal conditions. The esthetic subjective impact of malocclusion was assessed by means of the Orthodontic Aesthetic Subjective Impact Score - OASIS, whereas the malocclusion and the need for orthodontic treatment were assessed by means of the Dental Aesthetic Index (DAI) and the Index of Orthodontic Treatment Need-Aesthetic Component (IOTN-AC).

**Results:**

Prevalence of normative need for orthodontic treatment was 65.6% (n = 155), and prevalence of orthodontic esthetic subjective impact was 14.9%. The following variables showed significant association with esthetic subjective impact of malocclusion: female (p = 0.042; OR = 0.5; CI = 0.2-0.9), public school student (p = 0.002; OR = 6.8; CI = 1.9-23.8), maxillary overjet ≥ 4 mm (p = 0.037; OR = 1.7; CI = 1-3) and gingival smile ≥ 4 mm (p = 0.008; OR = 3.4; CI = 1.3-8.8).

**Conclusion:**

The normative need for orthodontic treatment overestimated the perceived need. Occlusal and sociocultural factors influenced the dissatisfaction of schoolchildren with their dentofacial appearance.

## Abstract (pt)

**Objetivo:**

o objetivo desse estudo foi avaliar a necessidade normativa de tratamento ortodôntico e os fatores que determinam o impacto subjetivo da má oclusão, em escolares brasileiros de 12 anos.

**Métodos:**

um total de 451 indivíduos (215 homens e 236 mulheres) foi selecionado aleatoriamente de escolas públicas e particulares de Juiz de Fora, Minas Gerais. Os dados coletados incluíam informações sociodemográficas e condições oclusais. O impacto estético subjetivo da má oclusão foi avaliado pelo *Orthodontic Aesthetic Subjective Impact Score* (OASIS). A avaliação da má oclusão e a necessidade de tratamento ortodôntico foram avaliadas pelo *Dental Aesthetic Index* (DAI) e pelo *Index of Orthodontic Treatment Need-Aesthetic Component* (IOTN-AC).

**Resultados:**

a prevalência da necessidade normativa de tratamento ortodôntico foi de 65,6% (n = 155) e a prevalência do impacto estético ortodôntico subjetivo foi de 14,9%. As seguintes variáveis mostraram associação significativa com impacto estético subjetivo da má oclusão: sexo feminino (p = 0,042, OR = 0,5, IC = 0,2-0,9); aluno de escola pública (p = 0,002, OR = 6,8, IC = 1,9-23,8); ≥ 4mm (p = 0,037, OR = 1,7; ICI = 1-3); e sorriso gengival ≥ 4mm (p = 0,008, OR = 3,4, IC = 1,3-8,8).

**Conclusão:**

a necessidade normativa de tratamento ortodôntico superestimou a necessidade percebida. Fatores oclusais e socioculturais influenciaram a insatisfação de escolares com a aparência dentofacial.

## INTRODUCTION

Malocclusion is a craniofacial growth and development disorder that may lead to functional problems with esthetic impact and consequent psychosocial implications in children and adults.^1,5^ It is considered a public health concern and the third most frequent oral disorder after dental caries and periodontal problems. Thus, orthodontists must include in diagnosis and planning, instruments that highlight the influence of sociocultural components and their relation to the perception of the malocclusion developed by the individual.

Efforts to develop solid diagnostic criteria that allow patients to understand their problems have been the main focus of dentists and orthodontists; however, it is difficult to determine the importance of malocclusion as a facial problem and its impact on the quality of life of the individuals affected.^6^ This problem is particularly more complex during childhood due to constant changes in psychosocial and body characteristics and great variability in cognitive development that occur in children of the same age group.^7^

Orthodontic treatment during childhood is generally associated with esthetic problems^8,9^ normally related to considerable diversity in patient's perception process.^7,10^ Although the esthetic impact of malocclusion greatly influences a child's biopsychosocial development,^11^ little attention has been given to its association with normative values of treatment needs.

This study aims at assessing the normative need for orthodontic treatment and the factors determining the subjective impact of malocclusion on 12-year-old Brazilian schoolchildren.

## MATERIAL AND METHODS

This cross-sectional study was carried out with 12-year-old Brazilian schoolchildren randomly selected from public and private schools of Juiz de Fora - Minas Gerais/Brazil. Cluster sampling method was used with proportional raffling of school categories: public (local, state and federal) and private. The participating classes and schoolchildren were also raffled.

Sample size (n = 451) was calculated based on demographic data from the "Brazilian oral health report - 2010"^12^ considering 38% of estimated malocclusion prevalence at the age of 12,^13^ with 95% confidence interval and 5% standard error.^14^

The following exclusion criteria were applied: Craniofacial malformation or syndromes with dentofacial manifestations, previous orthodontic treatment and mental or behavior disorders that could interfere in patient's self-perception of the assessed factors. This project was approved by the Institutional Review Board of the Federal University of Juiz de Fora. Patients' parents or guardians signed an informed consent form.

The collected data included sociodemographic information and clinical features concerning the subjects' occlusal conditions. The schoolchildren answered a questionnaire that included a test to assess the esthetic subjective impact of malocclusion (Orthodontic Aesthetic Subjective Impact Score - OASIS). Malocclusion as well as the need for orthodontic treatment were assessed through the Dental Aesthetic Index (DAI) and the Index of Orthodontic Treatment Need-Aesthetic Component (IOTN-AC). For economic characterization, patients' parents or guardians answered a self-applied questionnaire.^15^

### Dental examination

Dental examination followed the World Health Organization criteria for oral health research.^16^ All oral assessments were performed by the same orthodontist who was previously calibrated and trained for all indexes used in this study. Intra-observer agreement was calculated by Kappa coefficient (96%).

### Dental aesthetic index (DAI)

DAI assesses the esthetic aspects of dental occlusion, identifying the need for orthodontic treatment based on malocclusion severity. Its scale defines severity in a similar manner to orthodontists' judgment. DAI scores equal to or lower than 25 refer to malocclusions with a slight need for orthodontic treatment. Scores varying from 26 to 30 represent malocclusion with elective need for treatment. Scores varying from 31 to 35 represent malocclusion with a high need for treatment. Scores ≥ 36 represent severe malocclusion with compulsory need for treatment.^17^

### Orthodontic Aesthetic Subjective Impact Score - OASIS

OASIS measures the subjective aesthetic impact of malocclusion, assessing the degree of dissatisfaction of children with their teeth.^13^ OASIS has been validated and culturally adapted to Brazilian Portuguese.^18^ It comprises 5 questions, with the answers matching into the 7-point Likert scale. This dependent variable was dichotomized into satisfied or dissatisfied. Based on self-perception, the child was asked to identify in the IOTN-AC, the photography that best matched their oral condition. In order to facilitate children's understanding, the Likert scale was reduced to 3 possible answers; maintaining the initial and final scoring limits (5 - 35 points) of the original instrument, which were further added to the answers of the IOTN-AC (1 to 10). Median was used as the cut-off point to define aesthetic malocclusion impact. According to the IOTN-AC criteria, assessment of the need for orthodontic treatment classified the subjects into 3 groups: no need (1-4), borderline cases (5-7) and definite need (8-10).^19^

### Statistical analysis

Initially, a descriptive analysis of results was performed. Next, associations between dependent and independent variables were tested by means of the univariate analysis (chi-square test and Fisher's exact test) and both simple and multiple logistic regression analyses (stepwise forward procedure). The lack of an association between variables was considered as the null hypothesis (significance values greater than 0.05). The Statistical Package for the Social Sciences program (SPSS) - SPSS Inc., Chicago, USA, version 8.0, was used for statistical analysis.

## RESULTS

The total sample of 451 schoolchildren comprised 215 (47.7%) males and 236 (52.3%) females. With regard to skin color, 299 (66.3%) were white and 152 (33.7%) were classified as non-white. As for their economic level, 373 guardians (82.7%) adequately answered the questionnaire, thus yielding the following results: 8.6% - class A (n = 32); 32.2% - class B (n = 120); 45.2% - class C (n = 169); 13.7% - class D (n = 51); and 0.3% - class E (n = 1) (Table 1).

**Table 1:** Sample characterization according to sex, skin color, economic level and school category.

Sample characterization	Absolute frequency (n)	Relative frequency (%)
**Sex**		
Male	215	47,7
Female	236	52.3
**Skin color**		
White	299	66.3
Non-white	152	33.7
**Economic level**		
High (Class A and B)	152	40.8
Intermediate (Class C)	169	45.2
Low (Class D and E)	52	14.0
**School category**		
Private	126	27.9
Public	162	35.9
State	153	33.9
Federal	10	2.2

The normative need for orthodontic treatment and aesthetic subjective impact of malocclusion are presented in Table 2.

**Table 2:** Need for orthodontic treatment and aesthetic subjective impact of malocclusion in 12-year-old schoolchildren of Juiz de Fora/Minas Gerais.

Variables	Absolute frequency (n)	Relative frequency (%)
**Orthodontic treatment need (DAI)**		
No or slight need	155	34.4
Elective treatment	148	32.8
Highly desirable treatment	86	19.1
Compulsory need	62	13.7
**Orthodontic treatment need (IOTN-AC)**		
No need	362	80.3
Borderline cases	57	12.6
Definite need	32	7.1
**Aesthetic Subjective Impact of Malocclusion (OASIS)**		
Very satisfied	235	52.1
Satisfied	149	33.0
Dissatisfied	52	11.5
Very dissatisfied	15	3.4

Table 3 shows the results of the bivariate analysis for the dependent variable "aesthetic subjective impact of malocclusion" (OASIS), considering the occlusal alterations. Only maxillary overjet ≥ 4 mm, and gingival exposure at smile ≥ 4 mm were statistically associated with child's tooth appearance dissatisfaction.

**Table 3:** Association between occlusal alterations and appearance satisfaction of 12-year-old schoolchildren of Juiz de Fora/Minas Gerais.

Occlusal Alterations	Appearance Satisfaction (OASIS)				*Odds Ratio (95% CI)*	p
	No		Yes			
	(n)	(%)	(n)	(%)		
**Missing upper tooth**						
Not observed	62	14.6	364	85.4	1	0.457
Observed	5	20.0	20	80.0	1.4(0.5-4.0)	
**Missing lower tooth**						
Not observed	66	14.8	381	85.2	1	0.567
Observed	1	25.0	3	75.0	1.9 (0.1-18.7)	
**Incisor crowding**						
None	19	14.0	117	86.0	1	0.425
One or more segments	48	15.2	267	84.8	1.1 (0.6-1.9)	
**Incisor spacing**						
None	43	14.1	261	85.9	1	0.541
One or more segments	24	16.3	123	83.7	1.1 (0.6-2.0)	
**Median diastema**						
≤ 1 mm	58	14.2	351	85.8	1	0.209
≥ 2 mm	9	21.4	33	78.6	1.6 (0.7-3.6)	
**Maxillary malalignment**						
≤ 1 mm	39	13.7	245	86.3	1	0.382
≥ 2 mm	28	16.8	139	83.2	1.2 (0.7-2.1)	
**Mandibular malalignment**						
≤ 1 mm	36	13.0	241	87.0	1	0.161
≥ 2 mm	31	17.8	143	82.2	1.4 (08-2.4)	
**Maxillary overjet**						
≤ 3 mm	27	11.0	218	89.0	1	0.009
≥ 4 mm	40	19.4	166	80.6	1.9 (1.1-3.3)	
**Anterior crossbite**						
No	65	14.8	375	85.2	1	0.754
Yes	2	18.2	9	81.8	1.2 (0.2-6.0)	
**Anterior openbite (mm)**						
= 0 mm	63	14.5	372	85.5	1	0.245
≥ 1 mm	4	25.0	12	75.5	1.9 (0.6-6.2)	
**Molar relationship**						
Class I	20	12.0	147	88.0	1	
Class II	44	18.0	200	82.0	1.6 (0.9-2.8)	0.062
Class III	3	7.5	37	92.5	0.5 (0.1-2.1)	0.311
**Posterior crossbite**						
No	53	14.2	321	85.8	1	0.368
Yes	14	18.2	63	81.8	1.3 (0.7-2.5)	
**Gingival smile (mm)**						
≤ 3 mm	59	13.7	371	86.3	1	0.002
≥ 4 mm	8	38.1	13	61.9	3.8 (1.5-9.7)	

Regarding the relationship between the dependent variable and the sociodemographic variables, a statistically significant association was found for school category (p < 0.001), low economic level (p < 0.001), intermediate economic level (p = 0.004) and sex (p = 0.043) (Table 4).

**Table 4:** Association between socioeconomic variables (number and percentages) and appearance satisfaction of 12-year-old schoolchildren of Juiz de Fora/ Minas Gerais.

Sociodemographic variables	Appearance satisfaction (Oasis)				*Odds Ratio*	p
	No		Yes			
	(n)	(%)	(n)	(%)	(95% CI)	
**Skin color**						
Non-white	27	17.8	125	82.2	1	0.216
White	40	13.4	259	86.6	0.7 (0.4-1,2)	
**Sex**						
Female	42	17.8	194	82.2	1	0.043
Male	25	11.6	190	88.4	0.6 (0.3-1.0)	
**Economic level**						
High	11	7.2	141	92.8	1	0.004
Intermediate	30	17.8	139	82.2	2.7 (1.3-5.7)	<0.001
Low	13	25.0	39	75.0	11.9 (4.6-30.6)	
**School category**						
Private	4	3.2	122	96.8	1	0.000
Public	63	19.4	262	80.6	7.3 (2.6-20.6)	

Multiple logistic regression for occlusal and sociodemographic characteristics, which were significantly associated at bivariate analysis (p < 0.20), indicated the following variables as factors associated with the aesthetic subjective impact of malocclusion: female (p = 0.042; OR = 0.5; CI = 0.2-0.9), public school student (p = 0.002; OR = 6.8; CI = 1.9-23.8), maxillary overjet ≥ 4 mm (p = 0.037; OR = 1.7; CI=1-3) and gingival smile ≥ 4 mm (p = 0.008; OR = 3.4; CI = 1.3-8.8).

## DISCUSSION

The need for orthodontic treatment is difficult to be recognized by professionals, given that the decision on the need for orthodontic treatment must integrate clinical criteria and perceptible needs.^18,20,22^ Several authors report a tendency to overestimate the need for orthodontic treatment when normative criteria are used.^20,22,23^ The present results confirm these findings when DAI values are compared to the aesthetic subjective impact of malocclusion (OASIS). However, Marques et al^19^ found the opposite in a study conducted with Brazilian adolescents, probably due to the social status related to the use of orthodontic appliances and the free treatment offered by the public institution(as part of the Brazilian public health system) where the study was carried out.

Dental aesthetics has significant implications on an individual's quality of life and psychosocial relationships, being an important factor for those who seek orthodontic treatment.^3,5^ In this study, however, the aesthetic subjective impact of malocclusion (OASIS) was significantly (p < 0.001) lower than normative needs. Only 14.9% of individuals comprising the sample were not satisfied with their tooth appearance. Although the aesthetic subjective impact of malocclusion (OASIS) was strongly associated with normative impact (DAI), the difference in the frequencies found leads to important reflections on the need for orthodontic treatment. Once aesthetics is considered one of the most important aspects in orthodontic treatment,^3,5^ the instrument used to assess its impact should be more closely related to the self-perceived need for orthodontic treatment and appearance satisfaction than to normative indexes, only. Yet, they were significantly different in this study.

Normative need for treatment (DAI) was strongly associated with the aesthetic subjective impact of malocclusion (p = 0.007). However, when occlusal alterations were analyzed separately, only the variables overjet ≥ 4 mm and gingival smile ≥ 4 mm were statistically significant. Association between great overjet and gingival smile with appearance satisfaction has been reported in other studies.^7,24,25^ Increase in overjet is strongly associated with the risk of dental injuries due to lack of labial seal and exposure of upper teeth. Additionally, the social impact of this problem must be considered as an important factor for facial stigmatization. The same occurs when gingival smile is assessed.^5,7^ In the present study, these problems clearly affected patients' dental dissatisfaction.

Except for skin color, all other sociodemographic variables showed statistically significant association with the aesthetic impact of malocclusion. In agreement with other studies, this impact was more significant in females^23,26 ^and intermediate economic level. Yet, after logistic regression, only sex and school remained significantly associated with the impact of malocclusion.

Some occlusal conditions related to aesthetic impairment, such as incisor crowding, upper and lower misalignment and missing teeth, were not associated with the aesthetic subjective impact of malocclusion (OASIS). This finding was in disagreement with a previous study which showed an association between this instrument and occlusal alterations.^23^ This fact highlights the great variability and complexity of perception of facial aesthetics, with significant differences between normative and self-perceived values.^27,30^ The exclusive use of normative criteria to determine the need for orthodontic treatment does not consider the subjective aspects related to the individual's perception and the psychosocial implications of malocclusions. For this reason, it tends to overestimate the prevalence of malocclusion with treatment need. This is particularly important for the planning of health policies, especially in underfunded public health services.^6,20,21^

## CONCLUSIONS

- The normative need for orthodontic treatment overestimated the perceived need.
- The variables sex, school category, maxillary overjet ≥ 4 mm and gingival smile ≥ 4 mm negatively influenced patient's satisfaction with dentofacial appearance.
